# THz Generation by Two-Color Plasma: Time Shaping and Ultra-Broadband Polarimetry

**DOI:** 10.3390/s24134265

**Published:** 2024-06-30

**Authors:** Domenico Paparo, Anna Martinez, Andrea Rubano, Jonathan Houard, Ammar Hideur, Angela Vella

**Affiliations:** 1 ISASI—Institute of Applied Sciences and Intelligent Systems, Consiglio Nazionale delle Ricerche, via Campi Flegrei 34, 80078 Pozzuoli, Italy; anna.martinez@unina.it (A.M.); andrea.rubano.80@gmail.com (A.R.); 2 Dipartimento di Fisica “Ettore Pancini”, Università di Napoli “Federico II”, Complesso Universitario di Monte Sant’Angelo, via Cintia, 80126 Napoli, Italy; 3 Scuola Superiore Meridionale, Largo San Marcellino, 80138 Napoli, Italy; 4 University Rouen Normandie, INSA Rouen Normandie, CNRS, Normandie University, GPM UMR 6634, F-76000 Rouen, France; jonathan.houard@univ-rouen.fr (J.H.); angela.vella@univ-rouen.fr (A.V.); 5 CORIA CNRS, INSA, Université de Rouen Normandie, F-76801 Saint Etienne du Rouvray, France; hideur@coria.fr

**Keywords:** THz generation by two-color plasma, ultra-broadband THz polarimetry, THz vector beams, photocurrent model, second-harmonic generation in air

## Abstract

The generation of terahertz radiation via laser-induced plasma from two-color femtosecond pulses in air has been extensively studied due to its broad emission spectrum and significant pulse energy. However, precise control over the temporal properties of these ultra-broadband terahertz pulses, as well as the measurement of their polarization state, remain challenging. In this study, we review our latest findings on these topics and present additional results not previously reported in our earlier works. First, we investigate the impact of chirping on the fundamental wave and the effect of manipulating the phase difference between the fundamental wave and the second-harmonic wave on the properties of generated terahertz pulses. We demonstrate that we can tune the time shape of terahertz pulses, causing them to reverse polarity or become bipolar by carefully selecting the correct combination of chirp and phase. Additionally, we introduce a novel technique for polarization characterization, termed terahertz unipolar polarimetry, which utilizes a weak probe beam and avoids the systematic errors associated with traditional methods. This technique is effective for detecting polarization-structured terahertz beams and the longitudinal component of focused terahertz beams. Our findings contribute to the improved control and characterization of terahertz radiation, enhancing its application in fields such as nonlinear optics, spectroscopy, and microscopy.

## 1. Introduction

The emission of terahertz (THz) radiation from laser-induced plasma, created by focusing two-color femtosecond pulses in air or gas, is a widely utilized technique due to its ultra-wide emission spectrum and relatively high THz pulse energy and peak power [1,2,3,4,5,6]. The broad bandwidth enables ultra-broadband spectroscopic studies of various materials in the THz range, including phonon modes in molecular crystals [7], semiconductors [8], conductive oxides [9], chalcogenide glasses [10], photovoltaic materials [11], polymers [12], liquids [13,14,15], liquid crystals [16], crystalline diamonds [17], and biosystems [18]. Additionally, the high peak power of the generated THz pulses can induce nonlinear optical effects in crystalline materials [19,20] or assist in ion and electron emission in atom probe tomography [21].

Despite its significance, accurately characterizing and controlling the polarization and spatiotemporal properties of ultra-broadband THz pulses remains challenging, due to the lack of suitable optics. On the detection side, THz air-biased coherent detection has emerged as a promising solution, utilizing heterodyne detection and second-harmonic generation (SHG) induced by THz radiation [7,22]. However, recent research indicates that the laser-induced air plasma used in this technique can exhibit birefringence, leading to systematic errors in polarization-state determination [23].

In terms of control, a key aspect is optimizing the THz-optical conversion process through the chirp of the fundamental wave (FW), which affects the properties of the generated THz fields. Zhang et al. [24] investigated the influence of chirp on generated THz power, finding a non-monotonic dependence with two maxima and a local minimum. Similar findings were reported by Mou et al. [25]. They also explored the relationship between chirp and the polarization of the generated THz radiation. As we will show later, chirp can also significantly influence the THz temporal waveform. The latter property is crucial for applications where the polarity of the optical field is important, such as atom probe tomography [21], the orientational control of molecules [26], ferroelectric domain switching [27], and THz-assisted scanning tunneling microscopy [28].

In this paper, we review our latest results on controlling the temporal shape and polarization characterization of ultra-broadband THz pulses generated by two-color air plasma. Moreover, we will discuss additional results that have not previously been reported in our earlier works [29,30].

Regarding generation, we demonstrate that the chirp of the FW and the phase difference between the FW and the second-harmonic wave (SHW) are pivotal in governing the properties of the generated THz pulses, including their temporal shape and generation efficiency. Positive and negative chirps result in distinct pulse shapes. We demonstrate that we can tune the THz pulse shape to reverse its polarity or become bipolar by carefully selecting the correct combination of chirp and phase. Additionally, our findings indicate that the relationship between THz pulse energy and chirp varies with changes in the specific phase difference between FW and SHW. On this point, we report new experimental results that complete the data presented in Ref. [29].

For polarization characterization, we describe a simplified approach utilizing a weak probe beam and avoiding high-voltage DC bias fields. Unlike the terahertz air-biased coherent detection scheme (ABCD), our method yields a unipolar, intensity-proportional SHG signal; hence, we term this technique THz unipolar polarimetry (TUP). We will show that, unlike the ABCD technique, TUP is free from systematic errors and is highly effective in detecting the polarization state of ultra-broadband THz beams, including polarization-structured beams (THz vector beams). Moreover, in this article, we will present a new theoretical analysis that will extend the theory presented in Ref. [30] to include THz beams with a longitudinal component, as in the case of highly focused beams. We will demonstrate that the TUP technique is capable of measuring the axial component of such beams.

## 2. Theory

In the next two sections, we present the theoretical background needed to discuss the results shown in Section 4. In Section 2.1, we will focus on the theory behind the generation of THz pulses through the two-color technique in plasma, while in Section 2.2, we discuss the theory of THz-induced SHG in air, which is fundamental to the TUP technique. Although the roles of the THz field in Section 2.1 and Section 2.2 differ, these phenomena share the same underlying physics. Therefore, the basic equations governing both phenomena are very similar.

When addressing THz generation in two-color filamentation, it is essential to consider the intensity of the fundamental wave generating the plasma. In fact, depending on the intensity of the fundamental wave, we can distinguish two regimes [31]: photocurrent generation and four-wave mixing (FWM). The first model will be detailed in Section 1, while the second is described by the following equation [32]:

(1) $$E_{i}^{THz}\propto \chi_{ijlm}E_{j}^{2\omega }E_{l}^{\omega }E_{m}^{\omega }$$

where $\chi_{ijlm}$ is the third-order susceptibility tensor of plasma or air, depending on whether we are below the plasma-formation threshold; $E^{\omega }$ and $E^{2\omega }$ are the fundamental and second-harmonic optical fields, respectively. In the latter equation, the Einstein rule on repeated indices is applied.

**Figure 1:**
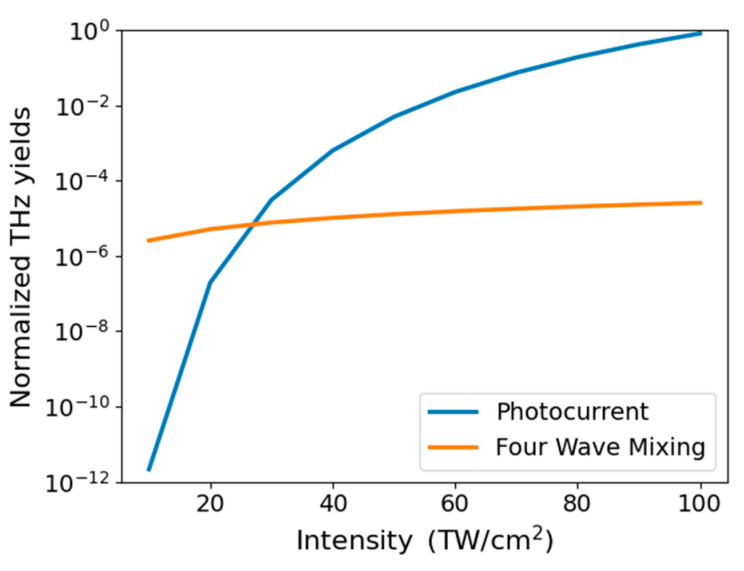
Intensity of the generated THz field as a function of the FW intensity of the two mechanisms: four-wave mixing (orange line) and plasma photocurrent (blue line). The values have been normalized to the maximum yield obtained with the photocurrent mechanism.

The competing strengths of these two regimes are illustrated in Figure 1, where we report the results of our photocurrent model. The simulations are based on the parameters detailed in Section 2.1, while for simulating the FWM contribution, we use a value for third-order susceptibility of $\approx 10^{-26}\;$(m^2^/V^2^), as reported in Ref. [31]. We note that our results are in line with the latter, pioneering work. As seen in Figure 1, at the lowest intensities, THz generation is dominated by the FWM process. However, as the FW intensity increases, the plasma photocurrent regime becomes predominant by several orders of magnitude. Incidentally, a direct measurement of photocurrent at this level of intensity has been reported in Ref. [33].

In our experimental setup, THz generation occurs primarily in the photocurrent regime. Therefore, the next section will focus on the photocurrent model. When applying TUP under the experimental conditions for detection, the FW intensity is very low. Consequently, as we will see, this scenario is better described by an equation that is very similar to Equation (1).

### 2.1. The Photocurrent Model

To understand the impact of chirp on THz signal generation, we utilize the photocurrent model described by Nguyen et al. [34]. Our primary focus is on describing the temporal dependence of the scalar electric field E(t), so we will consider neither the vectorial nature of the polarization nor the transverse intensity distribution of the pulse. Consequently, we will assume a linearly polarized plane wave throughout this discussion. According to the model, the THz field, $E_{THz}$, is proportional to the first derivative of the photocurrent J induced by free electrons:

(2) $$E_{THz}\left(r,\;t\right)=g\partial_{t}J\left(r,t\right),$$

where the geometrical factor *g* originates from Jefimenko’s theory [35]. In the following, we will apply local current approximation (LC), which implies that in Equation (2), *J* is approximated by a delta function of the z coordinate. This assumption implies that we neglect several effects due to propagation in plasma, appearing as chirp or phase variation (Gouy phase) around the lens focal point. Despite the simplicity of this model, we will show its capability of capturing the main qualitative features of our experimental outcomes. At moderate intensities (<10^15^ Wcm^−2^), the temporal shape of *J* follows a plasma fluid model, described by the following equation:

(3) $$\frac{dJ(t)}{dt}+v_{c}J\left(t\right)=\frac{e^{2}}{m_{e}}N_{e}\left(\left[E\left(t\right)\right],t\right)E\left(t\right).$$

Here, the letters e and $m_{e}$ represent the electron charge and mass, respectively, and $v_{c}\approx 1\;$ps^−1^ denotes the electron-neutral collision rate. The density of free electrons, $N_{e}$, is given by:

(4) $$\frac{dN_{e}(t)}{dt}=W\left(\left|E\left(t\right)\right|\right)\left(N_{a}-N_{e}\left(t\right)\right),$$

where *N*_a_ ≈ 2.16 × 10^19^ cm^−3^ represents the initial gas density [36], and *W*(|*E*(t)|) is the ionization rate. Under the approximation of the quasi-static tunneling model [4], the ionization rate is written as:

(5) $$W\left(\left|E\left(t\right)\right|\right)=\frac{KE_{au}}{\left|E(t)\right|}e^{-\frac{\beta E_{au}}{\left|E(t)\right|}},$$

where $K=4\omega_{au}r_{H}^{5/2}$, $\beta =2/3r_{H}^{5/2}$, $E_{au}=-m_{e}^{2}e^{5}/\hbar^{5}$, and $\omega_{au}=m_{e}e^{4}/\hbar^{3},$ while $r_{H}=$ $U_{ion}^{N}/U_{ion}^{H}$ is the ratio of the ionization potential of nitrogen ($U_{ion}^{N}$ = 15.6 eV) to hydrogen ($U_{ion}^{H}$ = 13.6 eV).

By combining Equations (1)–(3), we find, for the Fourier transform of the electric field:

(6) $${\hat{E}}_{THz}\left(\omega \right)=\frac{ge^{2}}{m_{e}\left(1+\frac{iv_{c}}{\omega }\right)}{\hat{N}}_{e}(\omega )*\hat{E}(\omega ),$$

where * is the convolution product and the hat symbol is the Fourier transform to the frequency domain.

Now we introduce the expression for the chirped FW [37]:

(7) $$E_{\omega_{0}}\left(t\right)=A_{\omega_{0}}\frac{\sqrt{1+iC}}{\sqrt{1+C^{2}}}\exp\left[-2\ln2\left(1+iC\right)\frac{t^{2}}{\tau^{2}}-i\omega_{0}t\right],\;$$

where *C* is the chirp parameter and $\omega_{0}$ is the carrier frequency corresponding to the carrier wavelength $\lambda_{0}$, with $\tau =\tau_{0}\sqrt{1+C^{2}}$ and $\tau_{0}$ being the FWHM durations of the stretched and transform-limited input laser pulse, respectively.

The field of the SHW is given by:

(8) $$E_{2\omega_{0}}=\frac{\sqrt{R}}{\left|E_{\omega_{0}}\left(t=0\right)\right|}E_{\omega_{0}}^{2}(t)e^{-i∆\phi },$$

where *R* represents the fraction of FW intensity converted into SHW intensity, and ∆ϕ accounts for the phase difference between the two waves due to their propagation in air from the crystal during second-harmonic generation at the plasma front. It is important to note that our model is local and does not consider any phase variation caused by propagation in plasma. Despite its simplicity, we will demonstrate that this model successfully captures the main features of our experimental observations. The total field to be used in Equation (6) is given by:

(9) $$E\left(t\right)=E_{\omega_{0}}\left(t\right)+E_{{2\omega }_{0}}\left(t\right).$$

**Figure 2:**
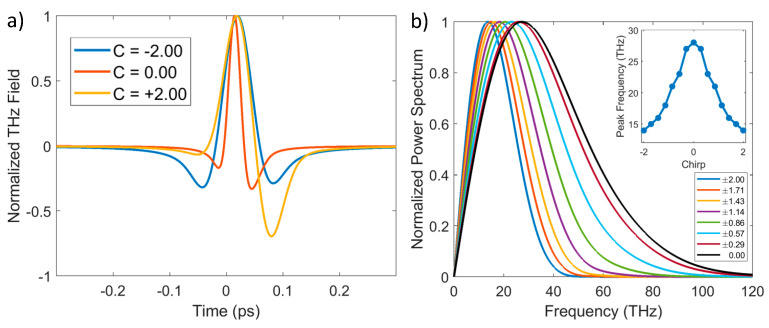
Panel (**a**) shows THz waveforms for different values of chirp. Panel (**b**) shows normalized power spectra for different chirp values. In the inset, the frequency of the maximum power as a function of the chirp is shown. For all graphs, ∆ϕ=7π/9.

This model can be applied to calculate the generated THz waveforms at different chirp and ∆ϕ values. Examples of these simulations are displayed in Figure 2. In the latter, we have used the following parameters: $\lambda_{0}=800$ nm, $I_{\omega_{0}}=10^{14}$ W/cm^2^, ∆ϕ=7π/9.

In Figure 2a, it is observed that the primary effect of the chirp is to lengthen the pulse duration, as expected. Naturally, this is accompanied by a narrowing of the spectra, as shown in Figure 2b. Another result of this spectral narrowing is a shift in the peak frequency, clearly illustrated in the inset of Figure 2b. An intriguing aspect of these simulations is the ability to use chirp to alter the time profile of the THz pulse. In Figure 2a, with a specific choice of ∆ϕ, the waveform becomes more bipolar for positive chirps. However, as we will demonstrate later, it is possible to alter the waveform as desired by appropriately adjusting the combination of chirp and ∆ϕ.

### 2.2. THz-Induced Second-Harmonic Generation in Air

**Figure 3:**
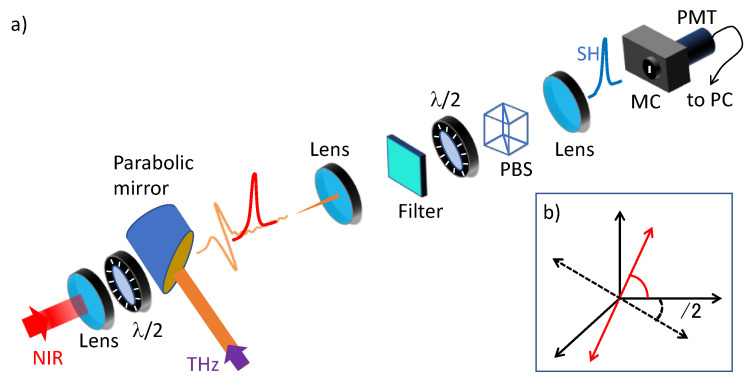
(**a**) Experimental scheme of the apparatus. FW and THz beams are collinearly focused on the same point, respectively, by a lens and a hole-drilled off-axis parabolic mirror. SHW is analyzed with a combination of a half-waveplate and a polarizing beam splitter (PBS), with the axis parallel to the $\hat{x}$ axis. A monochromator (MC) further rejects spurious signals, and a photomultiplier tube (PMT) measures the SHW intensity. Panel (**b**) shows the geometry of the different polarizations and the half-waveplate-analyzer axis: FW (red line); half-wave plate axis (dotted black line). [Reprinted from *Appl. Phys. Lett.* 2023, 1403923, 071101 [30], with the permission of AIP Publishing].

In this section, we present a comprehensive theory of the second-harmonic signal induced in air by a THz beam. We will thoroughly investigate the dependence of the SHW on the polarization state of the THz field, including THz fields with a longitudinal component or a spatially structured polarization state (vector beams). Let us consider the reference system depicted in Figure 3b and a linearly polarized fundamental beam, $E^{\omega }$, propagating in air, as shown in Figure 3a, and a generically polarized THz optical field, $E^{THz}$, propagating collinearly with the fundamental beam as shown in Figure 3a. The components for both beams are written as follows:

(10) Eω=E0cos⁡αE0sin⁡αiE0z; ETHz=abeiδic,

where *E*_0_ is the amplitude of the electric field in the plane $\hat{xy}$, perpendicular to the propagation direction, and α is the angle that the electric field forms with the $\hat{x}$ axis. In Equation (9), we have used the Jones vector notation for the THz optical field by introducing the phase δ between the $\hat{x}$ and $\hat{y}$ THz components [38]. Note that for Gaussian and Gaussian–Hermite beams, the axial component is generally 90° out of phase with the transverse component [39]. This fundamental point is emphasized in Equation (10) by explicitly indicating the imaginary unit and keeping the remaining quantities real for the simulations presented below. At the focal point of a suitable lens, the two beams of Equation (10) combine to generate a second harmonic of the fundamental beam according to the following expression which, as anticipated, is very similar to Equation (1):

(11) $$E_{i}^{2\omega }\propto \chi_{ijlm}E_{j}^{THz}E_{l}^{\omega }E_{m}^{\omega },$$

where $\chi_{ijlm}$ is the third-order susceptibility tensor of air. The third-order susceptibility of an isotropic medium, such as air, has only three independent elements, as shown in Table 1. We assume that these elements are real, i.e., the frequency of both the FW and its second harmonic are far from the resonances. Furthermore, these elements satisfy the following equation [32]:

(12) $${\chi_{4}\equiv \chi }_{xxxx}=\chi_{yyyy}=\chi_{zzzz}=\chi_{xxyy}+\chi_{yxyx}+\chi_{yxxy}.$$

Since air is an isotropic medium, we can choose the reference system arbitrarily. In Figure 3, we set the $\hat{z}$ axis parallel to the beam propagation and the $\hat{x}$ axis parallel to the axis of the polarizer used to analyze the polarization state of the second-harmonic beam. The FW polarization forms an angle with the $\hat{x}$-axis equal to α.

Making use of Table 1 and Equation (12), Equation (11) can be expanded to show that the two SHW transverse components have the following expressions:

(13) $$E_{x}^{2\omega }=E_{0}^{2}\left[\chi_{4}{E_{x}^{THz}\cos}^{2}\alpha +\chi_{1}{E_{x}^{THz}\sin}^{2}\alpha +\left(\chi_{2}+\chi_{3}\right)E_{y}^{\mathrm{THz}}\sin\alpha \cos\alpha +\left(\chi_{2}+\chi_{3}\right)E_{z}^{THz}\frac{E_{z0}}{E_{0}}\cos\alpha +\chi_{1}E_{x}^{THz}{\left(\frac{E_{z0}}{E_{0}}\right)}^{2}\right]$$

(14) $$E_{y}^{2\omega }=E_{0}^{2}\left[\chi_{1}{E_{y}^{THz}\cos}^{2}\alpha +\chi_{4}{E_{y}^{THz}\sin}^{2}\alpha +\left(\chi_{2}+\chi_{3}\right)E_{x}^{\mathrm{THz}}\sin\alpha \cos\alpha +\left(\chi_{2}+\chi_{3}\right)E_{z}^{THz}\frac{E_{0z}}{E_{0}}\sin\alpha +\chi_{1}E_{y}^{THz}{\left(\frac{E_{0z}}{E_{0}}\right)}^{2}\right]$$

In these expressions, it is interesting to note how the fourth terms on the right side depend on the longitudinal component, $E_{z}^{THz}$. Furthermore, these terms do not vanish, only if the FW has a longitudinal component along $\hat{z}$ too. A suitable measurement of these terms opens up the possibility of measuring the axial component of a THz field. By using Equation (10), Equations (13) and (14) can be written as follows:

(15) $$E_{x}^{2\omega }=E_{0}^{2}\left(\chi_{2}+\chi_{3}\right)\left[{a(\chi }_{a}\cos^{2}\alpha +\chi_{b}\sin^{2}\alpha )+be^{\mathrm{i\delta}}\sin\alpha \cos\alpha +ic\frac{E_{0z}}{E_{0}}\cos\alpha +{i\chi }_{b}c{\left(\frac{E_{0z}}{E_{0}}\right)}^{2}\right]\;$$

(16) $$E_{y}^{2\omega }=E_{0}^{2}\left(\chi_{2}+\chi_{3}\right)\left[{be^{i\delta }(\chi }_{b}\cos^{2}\alpha +\chi_{a}\sin^{2}\alpha )+a\sin\alpha \cos\alpha +ic\frac{E_{0z}}{E_{0}}\sin\alpha +\chi_{b}be^{i\delta }{\left(\frac{E_{0z}}{E_{0}}\right)}^{2}\right]\;$$

where, as we have written, $\chi_{a}=\chi_{4}/(\chi_{2}+\chi_{3})$ and $\chi_{b}=\chi_{1}/(\chi_{2}+\chi_{3})$. The amplitude of the SHW passing after the analyzer composed by the half-waveplate and the polarizer is given by:

(17) $$E_{2\omega }\left(\alpha ,\beta \right)=\cos\beta E_{x}^{2\omega }\left(\alpha \right)+\sin\beta E_{y}^{2\omega }(\alpha )$$

## 3. Methods

### 3.1. Experimental Set-Up for Controlling the Time-Shape of THz Pulses

**Figure 4:**
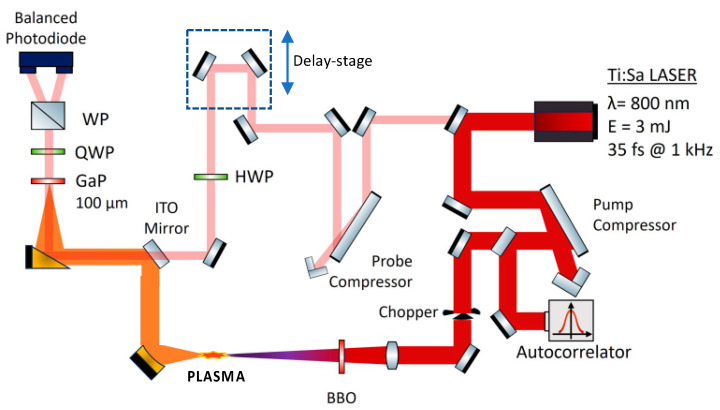
Schematic diagram of the experimental setup: HWP, half-waveplate; QWP, quarter-waveplate; WP, Wollaston prism. The pump beam passes through a BBO crystal to generate a second harmonic. Both beams are focused in air to form a plasma that produces a strong THz pulse. The probe beam is directed to the post-compression system to enable EO sampling. Note that the pump beam can be chirped independently of the probe beam.

This paper collects the results from two experiments conducted in separate laboratories. Despite the laser systems being acquired from different commercial providers, they produce pulses with remarkably similar characteristics. However, as shown in Figure 4, the apparatus detailed here includes two compression stages that enable independent manipulation of the pump and probe beams. This capability is crucial for adjusting the pump chirp while maintaining the constant timing characteristics of the probe beam used in electro-optic sampling.

For the experiment on time-shape control, we utilize a Ti:Sa laser system (Spectra Physics) that emits near-infrared pulses with a 35 fs duration at a central wavelength of 800 nm, operating at a repetition rate of 1 kHz and a maximum energy of 3 mJ per pulse. The laser beam is divided into two parts, with the higher-energy beam dedicated to THz generation. This higher-energy beam is mechanically chopped at a frequency of 500 Hz to increase the signal-to-noise ratio, as explained below.

As depicted in Figure 4, the beam is focused in air using a 30 cm focal length lens. Before reaching the focal plane, the laser beam passes through a beta-barium borate (BBO) crystal with a thickness of 100 µm. The latter component is positioned near the focus to reduce the impact of group-velocity dispersion effects. In the Supplementary Materials of Ref. [29], we demonstrate that this is the case in our geometry. The BBO crystal is mounted on a translation stage to vary Δϕ by exploiting air dispersion. To assign a specific value of Δϕ to each position of the BBO crystal, we follow the calibration procedure outlined in Ref. [4]. Further information on this procedure can be found in the Supplementary Materials of Ref. [29], where an estimate of the uncertainty of π/180 on ∆ϕ is also provided [29]. A lens combines the FW at a central wavelength of 800 nm, with its SHW in the focal point, generating a plasma filament that emits terahertz radiation with high field strength. This THz radiation is then directed onto the electro-optic sensing (EOS) system for further analysis. To minimize THz absorption by water vapor, a nitrogen environment is created from the plasma filament to the detection crystal.

The probe laser beam is compressed independently of the FW beam, as illustrated in Figure 4, and is used in the EOS stage. This stage comprises a 100 µm-thick gallium phosphide (GaP) crystal, a quarter-wave plate, and a Wollaston prism. The quarter-wave plate and the Wollaston prism project the probe polarization onto two circularly polarized states, which are then detected by two balanced photodiodes connected to a lock-in amplifier (Ametek’s 7265 Dual Phase) locked at the chopper frequency. This setup allows for precise measurements of the polarization changes induced in the probe by the THz field impinging on the GaP crystal. By adjusting the time delay between the pump and probe beams, a full reconstruction of the THz waveform is obtained. We induce chirp variation by adjusting the compressor stage of the laser system. The time duration of the pump pulse, measured using an autocorrelator (APE-pulseCheck), provides an estimate of the chirp. As shown in Figure 4, this autocorrelator is placed between the pump compressor and the chopper.

### 3.2. Apparatus for TUP Measurements

In this second apparatus, the broadband THz pulses are generated by means of the same technique in air-plasma that is described in Section 3.1. Here, the FW beam is provided by a similar laser system that was acquired from Coherent (Legend). A small part of the FW beam is used as a probe beam for detecting the THz pulse. The laser generates pulses with a central wavelength of approximately 800 nm, a pulse duration of about 35 fs, an output power of around 3.7 W, and a repetition rate of 1 kHz.

As shown in Figure 3a, the THz beam is focused by a parabolic mirror. For the calibration performed in the TUP technique, consisting of the measurement of the parameters $\chi_{a}$ and $\chi_{b}$ in Equations (15) and (16), the THz polarization is set in the $\hat{x}$ direction using a wire grid polarizer from TYDEX [40]. This polarizer is constructed on a polypropylene substrate, which naturally limits the initial bandwidth of the THz pulse to the 0.15–20 THz range. The focused probe beam passes through the hole of an off-axis parabolic mirror to propagate collinearly with the THz beam.

The generated SHW encounters a low-pass filter that rejects most of the fundamental probe beam. The SHW is then analyzed with a combination of a half-wave plate and a polarizing beam splitter (PBS). The selected SHW signal is further filtered by a monochromator and then detected by a photomultiplier tube (PMT). The PMT output signal is analyzed by a gated integrator to increase the signal-to-noise ratio and is eventually acquired by a computer.

## 4. Results and Discussion

### 4.1. Time-Shaping by Pulse Chirp and FW-SHW Frequency Dispersion

**Figure 5:**
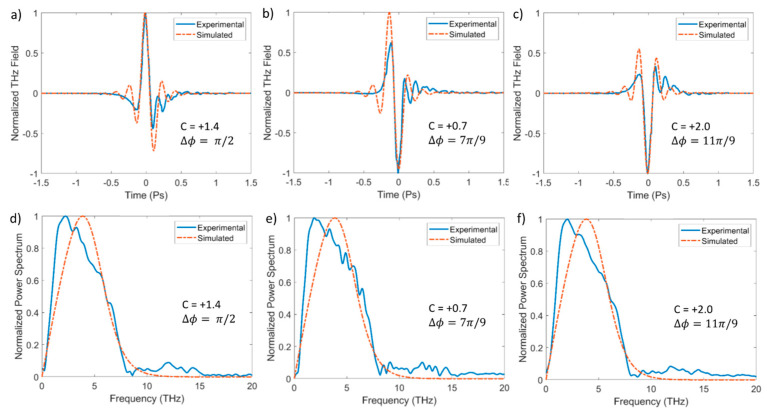
In panels (**a**–**c**), we present the measured (blue solid line) and simulated (red dashed line) THz waveforms for different values of chirp (positive) and ∆ϕ. In panels (**d**–**f**), the corresponding power spectra are shown. [Partly adapted from *Appl. Phys. Lett.* 2024 [29], 124, 021105, with the permission of AIP Publishing].

**Figure 6:**
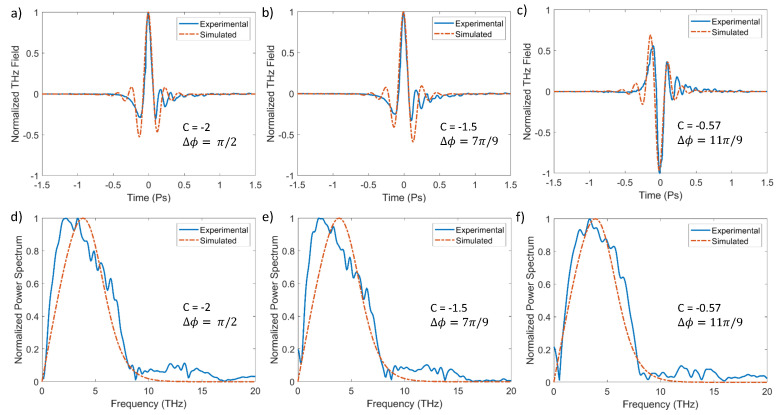
In panels (**a**–**c**), we present the measured (blue solid line) and simulated (red dashed line) THz waveforms for different values of chirp (negative) and ∆ϕ. In panels (**d**–**f**), the corresponding power spectra are shown. [Partly adapted from *Appl. Phys. Lett.* 2024, 124, 021105 [29], with the permission of AIP Publishing.

In Figure 5a–c and Figure 6a–c, we present examples of the measured electro-optic traces (blue lines) obtained in a nitrogen environment for positive and negative chirps, respectively. For each fixed BBO crystal configuration, we systematically varied the chirp, exploring both the positive and negative values. By acting on the pump compressor (see Figure 4), we can generate positively or negatively chirped pump pulses with a maximum duration of 80 fs. From the electro-optic traces, it is evident that a variation in chirp has a significant impact on the shape of the generated THz signal, as already highlighted in Figure 2. Note that the correlation between the chirp and ∆ϕ that is required to experimentally achieve a specific waveform is complex, also considering that the chirp can be controlled less precisely than ∆ϕ. Here, we present a selection of data that exemplarily demonstrates the polarity-inversion transition. Figure 5a–c and Figure 6a–c show the corresponding simulations (red dashed line) that were obtained using the photocurrent model described in Section 2.1. It is noteworthy that there is a good agreement between the experimental and theoretical results, despite the approximations of our model.

However, there is a noticeable discrepancy between the simulations shown in Figure 2 and the results presented in Figure 5. This discrepancy is further highlighted by the corresponding spectra in Figure 5d–f, which do not exhibit the same significant changes as are seen in Figure 2b and its inset. This difference arises because we cannot measure the full spectrum of the generated THz pulses and are limited by the bandwidth of the electro-optic crystal. GaP crystals are effective up to 8 THz, with a decline around 4 THz. To simulate the GaP response and achieve a meaningful comparison with the measured waveforms, in the simulations of Figure 5, we applied a cutoff at 8 THz. This accounts for the minimal variability in the calculated spectra. In the future, it would be interesting to conduct these experiments with a much broader detection bandwidth to verify the predictions suggested by Figure 2b. The same considerations apply to the data in Figure 6.

**Figure 7:**
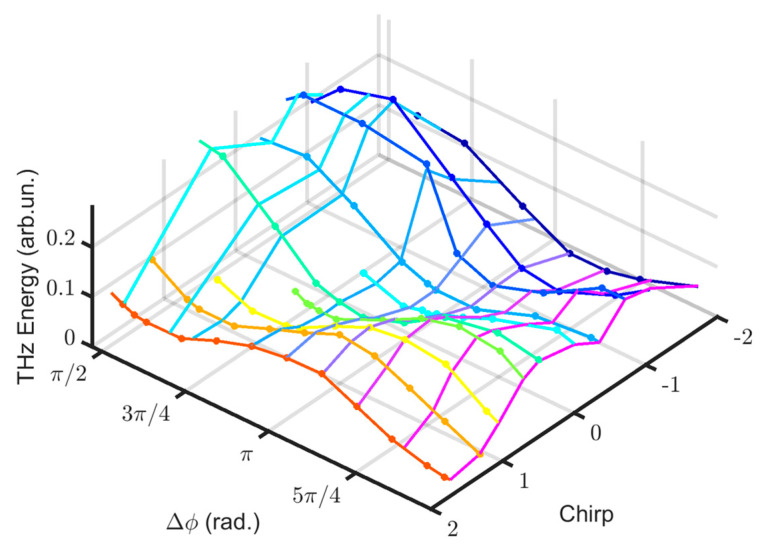
The energy of the generated THz pulses is shown as a function of ∆ϕ and chirp. The different colors correspond to different sets of measurements.

Commencing with the waveforms identified through the electro-optic system, we can perform a temporal integration of the square of the optical field magnitude. This process yields values that exhibit proportionality to the optical energy within the THz pulse. Figure 7 illustrates these values as a function of the phase shift angle and the chirp. For various phase shifts between FW and SHW, the shape of the THz energy optimization curve changes. For ∆ϕ values slightly greater than or equal to π/2, the THz energy optimization curve has its maximum for negative chirp; for ∆ϕ values of around π, the curve has its maximum for positive chirp; for ∆ϕ values slightly less than 3π/2, the curve presents two peaks at approximately the same energy for both positive and negative chirps. These results align well with other experiments documented in the literature [24,25,41]. Ultimately, we note that the pulse energy diminishes toward zero as the chirp increases on both sides, underscoring the impact of pulse lengthening on the FW and, consequently, SHW peak intensities.

### 4.2. THz Unipolar Polarimetry

The knowledge of parameters $\chi_{a}$ and $\chi_{b}$ in Equations (15) and (16) allows for the measurement of any arbitrary polarization of the THz pulse, including optical fields with longitudinal components. It is noteworthy that since detection occurs in air, the detection bandwidth of this method is theoretically unlimited, facilitating the reconstruction of the polarization state of ultra-broadband THz pulses.

The experimental determination of $\chi_{a}$ and $\chi_{b}$ can be achieved with high accuracy in the absence of fields with longitudinal components, thereby considering only the first three terms on the right-hand side of Equations (15) and (16). This requires setting a THz field with linear polarization parallel to the $\hat{x}$ axis and scanning the second-harmonic signal while varying the angles α and β of the half-waveplates as shown in Figure 3a.

**Figure 8:**
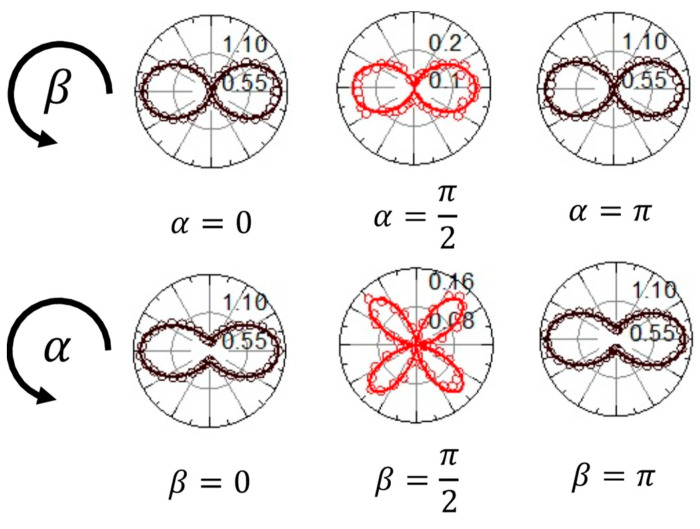
In the upper row of the figure, the plots show the quantity $I_{2\omega }(\alpha ,\beta )$ as a function of β for various values of α. The data points are represented by open circles, while the solid lines correspond to the fitting curves, as described in the main text. In the lower row of the figure, the plots depict the quantity $I_{2\omega }(\alpha ,\beta )$ as a function of α for various values of β. [Adapted from *Appl. Phys. Lett.* 2023, 123, 071101 [30], with the permission of AIP Publishing].

Figure 8 presents examples of these scans, along with the corresponding fits (solid lines) obtained using Equations (15)–(17). Except for an overall scale factor, the only adjustable parameters are $\chi_{a}$ and $\chi_{b}$. The excellent agreement between the data and the fitting curves also confirms the absence of systematic errors due to the use of intense probe beams, contrary to what was observed using the air-based coherent detection technique [23]. Fitting the data in Figure 8 results in $\chi_{a}=1.82\pm 0.06$ and $\chi_{b}=0.72\pm 0.03$.

**Figure 9:**
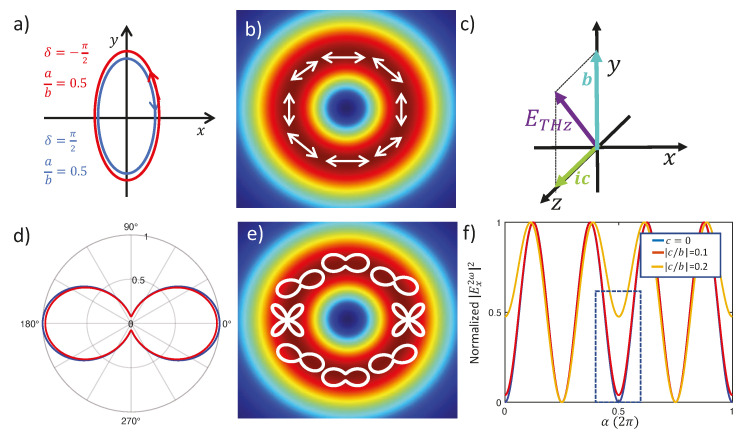
Panels (**a**–**c**) indicate the polarization states of the THz beam (see the explanation in the main text). Panels (**d**–**f**) show the corresponding normalized intensity distributions of the SHW. In panel (**d**), the blue curve has been slightly rescaled for clarity; actually, the two interferograms overlap exactly. In panel (**f**), $I\left(\alpha ,0\right)$ is calculated for different values of the ratio between the longitudinal, *c*, and the transverse, *b*, components. The blue dotted square highlights the values for α=π, where the curves show significant differences, to allow measurement of the axial component.

Once the technique is calibrated by measuring $\chi_{a}$ and $\chi_{b}$, it becomes possible to measure any polarization state of the THz beam, including beams with a longitudinal component or vector beams. For vector beams, the fundamental probe beam must be much smaller than the waist of the THz beam to scan it over the transverse section and reconstruct the polarization state point by point. Figure 9 theoretically discusses a few examples. Figure 9a–c indicates the polarization states of the THz beam: (a) an elliptically polarized THz beam with opposite handedness; (b) an azimuthal THz vector beam with a polarization pattern superimposed on the intensity distribution, characterized by a typical central singularity; (c) a linearly polarized beam with a longitudinal component. Note the imaginary unit to indicate that the transverse and longitudinal components are 90° out of phase. Below each panel, the corresponding intensity distribution of the SHW, $I\left(\alpha ,0\right)={\left|E_{2\omega }(\alpha ,0)\right|}^{2}$, is calculated from Equations (15) and (17). In Figure 9d, the blue curve has been slightly rescaled for clarity, but the two curves overlap perfectly. This illustrates a limitation of the technique, which, in its simplest form based on intensity measurements, cannot detect the handedness of elliptical polarization. However, Ref. [30] proposes a slight modification to the basic technique to recover the capability of measuring the wave’s phase and, thus, reconstructing the handedness.

In Figure 9e, the intensity distribution corresponding to each point of the vector beam is represented by white lines in the polar plots. Finally, Figure 9f shows interferograms for the different values of the ratio between the longitudinal and transverse components of linear polarization. As mentioned earlier, detecting longitudinal polarization requires using a fundamental beam with a longitudinal component. In all graphs in Figure 9f, the ratio between the transverse and longitudinal components of the fundamental wave is set at one. Although this assumption is physically reasonable, we might expect the longitudinal component to be much weaker than the transverse one. However, this is not necessarily true and strongly depends on the specific Gaussian–Hermite beam used and its position in the transverse section. In some cases, the longitudinal component can be significantly higher than the transverse component [39]. The blue line represents the perfectly symmetrical interferogram that is obtained when the longitudinal component vanishes. The interferogram becomes asymmetrical with the presence of a longitudinal component. Specifically, the signal for α=π becomes appreciably different from zero, even when the longitudinal component is only 1% of the transverse component, as highlighted by the blue dotted box in Figure 9f. This indicates that the two interferograms could be easily distinguishable using a null method. These findings demonstrate the technique’s capability to detect THz beams with a relatively small longitudinal component.

## 5. Conclusions

This article reviews our latest results on the generation and characterization of ultra-broadband terahertz pulses produced by laser-induced plasma in air using two-color femtosecond pulses. By examining the effects of chirping the fundamental wave and adjusting the phase difference between the FW and the SHW, we have demonstrated the ability to tailor the temporal profiles of THz pulses.

Furthermore, we introduced the THz unipolar polarimetry technique, which effectively measures the polarization state of ultrabroadband THz pulses, including those with longitudinal components. This method, which leverages a weak probe beam, circumvents the systematic errors associated with more intense probe beams, offering a more accurate approach to polarization detection.

Our results enhance the understanding and control of THz pulse properties, paving the way for more precise applications in nonlinear optics, spectroscopy, and microscopy. The ability to manipulate and accurately characterize THz radiation will likely lead to advancements in various scientific and technological fields, where the unique properties of THz waves are leveraged for probing and imaging.

## Figures and Tables

**Table 1:** Form of the third-order susceptibility tensor in air. Note the new symbols $\chi_{1}$, $\chi_{2}$, and $\chi_{3}$, introduced to simplify notation.

Isotropic Medium: 21 Elements; 3 Independent
${\chi_{1}\equiv \chi }_{yyzz}=\chi_{zzyy}=\chi_{zzxx}=\chi_{xxzz}=\chi_{xxyy}=\chi_{yyxx}$
$\chi_{2}\equiv \chi_{yzyz}=\chi_{zyzy}=\chi_{zxzx}=\chi_{xzxz}=\chi_{xyxy}=\chi_{yxyx}$
${\chi_{3}\equiv \chi }_{yzzy}=\chi_{zyyz}=\chi_{zxxz}=\chi_{xzzx}=\chi_{xyyx}=\chi_{yxxy}$
${\chi_{4}\equiv \chi }_{xxxx}=\chi_{yyyy}=\chi_{zzzz}=\chi_{xxyy}+\chi_{yxyx}+\chi_{yxxy}$

## Data Availability

The data that support the findings of this study are available from the corresponding author upon reasonable request.

## References

[1] Cook D., Hochstrasser R. (2000). Intense terahertz pulses by four-wave rectification in air. Opt. Lett..

[2] Bartel T., Gaal P., Reimann K., Woerner M., Elsaesser T. (2005). Generation of single-cycle THz transients with high electric-field amplitudes. Opt. Lett..

[3] Kim K.Y., Glownia J.H., Taylor A.J., Rodriguez G. (2007). Terahertz emission from ultrafast ionizing air in symmetry-broken laser fields. Opt. Express.

[4] Roskos H.G., Thomson M.D., Kreß M., Loeffler T. (2007). Broadband THz emission from gas plasmas induced by femtosecond optical pulses: From fundamentals to applications. Laser Photonics Rev..

[5] Koulouklidis A.D., Gollner C., Shumakova V., Fedorov V.Y., Pugzlys A., Baltuska A., Tzortzakis S. (2020). Observation of extremely efficient terahertz generation from mid-infrared two-color laser filaments. Nat. Commun..

[6] Buldt J., Stark H., Mueller M., Grebing C., Jauregui C., Limpert J. (2021). Gas plasma-based generation of broadband terahertz radiation with 640 mW average power. Opt. Lett..

[7] Ho I.C., Guo X., Zhang X.-C. (2010). Design and performance of reflective terahertz air-biased-coherent detection for time-domain spectroscopy. Opt. Express.

[8] Ho I.C., Zhang X.-C. (2014). Application of broadband terahertz spectroscopy in semiconductor nonlinear dynamics. Front. Optoelectron..

[9] Wang T., Zalkovskij M., Iwaszczuk K., Lavrinenko A.V., Naik G.V., Kim J., Boltasseva A., Jepsen P.U. (2015). Ultrabroadband terahertz conductivity of highly doped ZnO and ITO. Opt. Mater. Express.

[10] Zalkovskij M., Bisgaard C.Z., Novitsky A., Malureanu R., Savastru D., Popescu A., Jepsen P.U., Lavrinenko A. (2012). Ultrabroadband terahertz spectroscopy of chalcogenide glasses. Appl. Phys. Lett..

[11] Cooke D.G., Krebs F.C., Jepsen P.U. (2012). Direct Observation of Sub-100 fs Mobile Charge Generation in a Polymer-Fullerene Film. Phys. Rev. Lett..

[12] D’Angelo F., Mics Z., Bonn M., Turchinovich D. (2014). Ultra-broadband THz time-domain spectroscopy of common polymers using THz air photonics. Opt. Express.

[13] Wang T., Klarskov P., Jepsen P.U. (2014). Ultrabroadband THz Time-Domain Spectroscopy of a Free-Flowing Water Film. IEEE Trans. Terahertz Sci. Technol..

[14] Mou S., Rubano A., Paparo D. (2014). Broadband Terahertz Spectroscopy of Imidazolium-Based Ionic Liquids. J. Phys. Chem. B.

[15] Mou S., Rubano A., Paparo D. (2017). Complex Permittivity of Ionic Liquid Mixtures Investigated by Terahertz Time-Domain Spectroscopy. J. Phys. Chem. B.

[16] Vieweg N., Fischer B.M., Reuter M., Kula P., Dabrowski R., Celik M.A., Frenking G., Koch M., Jepsen P.U. (2012). Ultrabroadband terahertz spectroscopy of a liquid crystal. Opt. Express.

[17] Amoruso S., Andreone A., Bellucci A., Koral C., Girolami M., Mastellone M., Mou S., Orlando S., Papari G.P., Paparo D. (2020). All-carbon THz components based on laser-treated diamond. Carbon.

[18] Kumar R., Paturzo M., Sardo A., Orefice I., Yu Q., Rubano A., Paparo D. (2022). Toxic Effect of Metal Doping on Diatoms as Probed by Broadband Terahertz Time-Domain Spectroscopy. Molecules.

[19] Rubano A., Mou S., Marrucci L., Paparo D. (2019). Terahertz Hyper-Raman Time-Domain Spectroscopy. ACS Photonics.

[20] Mou S., Rubano A., Paparo D. (2019). Terahertz Hyper-Raman Time-Domain Spectroscopy of Gallium Selenide and its application in THz detection. Appl. Phys. Lett..

[21] Vella A., Houard J., Arnoldi L., Tang M., Boudant M., Ayoub A., Normand A., Costa G., Hideur A. (2021). High-resolution terahertz-driven atom probe tomography. Sci. Adv..

[22] Karpowicz N., Dai J., Lu X., Chen Y., Yamaguchi M., Zhao H., Zhang X.C., Zhang L., Zhang C., Price-Gallagher V. (2008). Coherent heterodyne time-domain spectrometry covering the entire ‘terahertz gap’. Appl. Phys. Lett..

[23] Zhang J. (2014). Polarization-dependent study of THz air-biased coherent detection. Opt. Lett..

[24] Zhang Z., Panov N., Andreeva V.A., Zhang Z., Slepkov A., Shipilo D., Thomson M.D., Wang T.-J., Babushkin I., Demircan A. (2018). Optimum chirp for efficient terahertz generation from two-color femtosecond pulses in air. Appl. Phys. Lett.

[25] Mou S., Tomarchio L., D’Arco A., Di Fabrizio M., Macis S., Curcio A., Palumbo L., Lupi S., Petrarca M. (2023). Impact of laser chirp on the polarization of terahertz from two-color plasma. Photonics Res..

[26] Machholm M., Henriksen N. (2001). Field-free orientation of molecules. Phys. Rev. Lett..

[27] Qi T., Shin Y.-H., Yeh K.-L., Nelson K.A., Rappe A.M. (2009). Collective coherent control: Synchronization of polarization in ferroelectric PbTiO3 by shaped THz fields. Phys. Rev. Lett..

[28] Yoshida S., Arashida Y., Hirori H., Tachizaki T., Taninaka A., Ueno H., Takeuchi O., Shigekawa H. (2021). Terahertz scanning tunneling microscopy for visualizing ultrafast electron motion in nanoscale potential variations. ACS Photonics.

[29] Martinez A., Houard J., Hideur A., Vella A., Paparo D. (2024). Controlling the time shape of terahertz pulses from two-color plasma by combining wavelength dispersion and laser chirp. Appl. Phys. Lett..

[30] Mou S., Rubano A., Yu Q., Paparo D. (2023). Terahertz unipolar polarimetry by second-harmonic generation in air. Appl. Phys. Lett..

[31] Bergé L., Skupin S., Koehler C., Babushkin I., Herrmann J. (2013). 3D Numerical Simulations of THz Generation by Two-Color Laser Filaments. Phys. Rev. Lett..

[32] Boyd R.W. (2008). Nonlinear Optics.

[33] Kim K.Y., Taylor A.J., Glownia J.H., Rodriguez G. (2008). Coherent control of terahertz supercontinuum generation in ultrafast laser-gas interactions. Nat. Photonics.

[34] Nguyen A., de Alaiza Martínez P.G., Thiele P., Skupin S., Bergé L. (2018). THz field engineering in two-color femtosecond filaments using chirped and delayed laser pulses. New J. Phys..

[35] Jefimenko O. (1966). Electricity and Magnetism: An Introduction to the Theory of Electric and Magnetic Fields.

[36] Amico C.D., Houard A., Akturk S., Liu Y., Bloas J.L., Franco M., Prade B., Couairon A., Tikhonchuk V.T., Mysyrowicz A. (2008). Forward thz radiation emission by femtosecond filamentation in gases: Theory and experiment. New J. Phys..

[37] Koulouklidis A., Fedorov V., Tzortzakis S. (2016). Spectral bandwidth scaling laws and reconstruction of THz wave packets generated from 2-color laser plasma filaments. Phys. Rev. A.

[38] Saleh B.E.A., Teich M.C. (2019). Fundamentals of Photonics.

[39] Novotny L., Hecht B. (2012). Principles of Nano-Optics.

[40] https://www.tydexoptics.com/products/thz_polarizers/thz_polarizers1/.

[41] Flender R., Sarosi K., Petracs E., Borzsonyi A., Chikan V. (2019). Control of THz field waveform emitted from air plasma by chirping two-color laser pulses. Opt. Commun..

